# Integration of transcriptome and metabolome analyses reveals key lodging-resistance-related genes and metabolic pathways in maize

**DOI:** 10.3389/fgene.2022.1001195

**Published:** 2022-10-10

**Authors:** Lei Liu, Songtao Liu, Haibo Lu, Zaimin Tian, Haichao Zhao, Dong Wei, Shuo Wang, Zhihong Huang

**Affiliations:** ^1^ Hebei Key Laboratory of Quality and Safety Analysis-Testing for Agro-Products and Food, Hebei North University, Zhangjiakou, China; ^2^ School of Medicine, Nankai University, Tianjin, China

**Keywords:** *Zea mays* L., RNA sequencing, metabonomics, lodging-resistance, microscopic structure

## Abstract

Stalk lodging, or breakage of the stalk at or below the ear, is one of the vital factors causing substantial yield losses in maize (*Zea mays*. L). Lodging affects maize plants’ physiological and molecular processes, eventually impacting plant growth and productivity. Despite this known fact, few researchers have investigated the genetic architecture underlying lodging in maize. Herein, through integrated transcriptome, metabolome, and phenotypic analyses of stalks of three diverse hybrid cultivars (highly resistant JNK738, mildly resistant JNK728, and lowly resistant XY335) at the tasseling (10 days to silking, 10 DTS) stage, we identified key genes and metabolic pathways modulating lodging resistance in maize. Based on the RNA-Seq analysis, a total of 10093 differentially expressed genes (DEGs) were identified from the comparison of the three varieties in pairs. Additionally, key lodging resistance–related metabolic pathways were obtained by KEGG enrichment analysis, and the DEGs were found predominantly enriched in phenylpropanoid and secondary metabolites biosynthesis pathways in the L_vs._H and M_vs._H comparison groups. Moreover, K-means analysis clustered the DEGs into clear and distinct expression profiles for each cultivar, with several functional and regulatory genes involved in the cell wall assembly, lignin biosynthetic process and hormone metabolic process being identified in the special clusters related to lodging resistance. Subsequently, integrating metabolome and transcriptome analyses revealed nine key lignin-associated metabolites that showed different expression trends in the three hybrid cultivars, among which L-phenylalanine and p-coumaric acid were regarded as differentially changed metabolites (DCMs). These two DCMs belonged to phenylalanine metabolism and biosynthesis pathways and were also supported by the RNA-Seq data. Furthermore, plant hormone signal transduction pathway–related genes encoding auxin, abscisic acid, jasmonates, and salicylic acid were differentially expressed in the three comparisons of lodging resistance, indicating these DEGs were valuable potential targets for improving maize lodging resistance. Finally, comparative physiological and qRT-PCR analyses results supported our transcriptome-based findings. Our research not only provides a preliminary theoretical basis and experimental ideas for an in-depth study of the regulatory networks involved in maize lodging resistance regulation but also opens up new avenues for molecular maize stalk lodging resistance breeding.

## Introduction

Stalk lodging, which refers to the permanent displacement of the stem from the vertical axis, has been a common problem in most cereals and other crops (Larsson et al., 2017). Several factors, including genetics, environmental conditions, field management, and insect infestation, can result in crop stalk lodging (Ennos et al., 1993; Esechie et al., 2004). In addition to reducing yield, lodging can significantly diminish the quality and efficiencies of production and mechanical harvesting processes. More scuttling and climate change–driven extreme weather events such as heavy rainfalls and strong winds exacerbate stalk lodging associated crop yield losses (Kumar et al., 2020)^.^

Maize (*Zea mays* L.) is a major cereal crop used globally as a source of food, animal feed, and fuel (Song et al., 2017). Additionally, it serves as an essential raw material in the food processing and beverage manufacturing industries. In addition, it is a vital experimental model organism (Aslam et al., 2015). In terms of global significance, maize is ranked amongst the three top most important cereal crops, together with rice (*Oryza sativa* L.) and wheat (*Triticum aestivum* L.) (Opitz et al., 2016). However, globally, maize production systems often encounter the problem of stalk lodging, which can seriously decrease crop photo-assimilation and overall photosynthesis, consequently reducing maize yield by approximately 5%–20% annually (Flint-Garcia et al., 2003; Ishimaru, 2004). During the plant’s vegetative growth stage, the rapid growth of the internodes weakens cell walls, increasing the probability for the stalk to break when strong winds are encountered (Ching et al., 2010). This is of major concern, particularly in the intensive maize production systems of Hebei Province of Northern China, where maize is grown under high plant densities, and the crop often encounters strong winds in autumn, thereby threatening grain yield. In addition to yield loss, stalk lodging increases harvest costs. In view of the China maize production system, which has embraced mechanical grain harvesting as the new development direction (Li et al., 2017), increasing maize stalk lodging resistance is vital for maintaining higher yields and reducing harvesting costs. Therefore, a detailed understanding of the genetic architecture of stalk lodging is important for successful breeding and biotechnological interventions aimed at enhancing grain yield and biomass quality.

Accurate evaluation of stalk lodging resistance in maize has been challenging because of influences of environmental factors that are not easy to control or replicate and difficulties in determining the aspects of stalk strength that translate into higher lodging resistance under field conditions. Several methods have been devised for the prediction of stalk strength, including measuring stalk crushing strength (SCS), stalk bending strength, and rind penetrometer resistance (RPR) (Nigam et al., 1974). Compared to other approaches, the RPR method has numerous advantages, including its feasibility and efficiency of operation, which has made it the method of choice for measuring stalk strength for the past century (Khanna, 1935; Flint-Garcia et al., 2003).

Meanwhile, several characteristics influence stalk lodging resistance of maize, including the thickness of stem cell walls, the total area of vascular bundles, and the vascular bundle area in the peripheral layer (Berzonsky et al., 1986; Islam et al., 2007; Zhang et al., 2018). Plants’ sclerenchyma cells have a thickened secondary cell wall which affects the strength of tissues. Therefore, increasing cell wall thickness is vital for enhancing stalk strength. Additionally, due to its role in cell rigidity, lignin content has been shown to affect stalk lodging (Pedersen et al., 2005). Therefore, lignin, as one of the main components in vascular plants, contributes to the mechanical strength of crop stems and is a potential target for enhancing stalk strength in plants (Wei et al., 2017).

Currently, efforts to reveal the molecular mechanisms regulating stalk lodging resistance of maize have been intensified. With quantitative trait loci (QTLs) analysis, a range of potential candidate genes involved in the biosynthesis of lignin was identified in maize, illustrating the complex nature of stalk strength (Hu et al., 2013; Li et al., 2014). The expression of *caffeic O-methyl transferase* (*COMT*) genes has been reported to improve the stalk lodging resistance of wheat by increasing stem rigidity (Vignols et al., 1995). It was shown that the decrease in lignin content and lodging resistance of maize mutant *bm1* was due to COMT inhibiting the synthesis of lignin monomers (Vignols et al., 1995). Several plant hormone–related genes are also involved in determining plant height. For instance, a set of genes influence plant height by regulating gibberellin (GA) biosynthesis and transport, including *dwarf1, dwarf3, dwarf plant 8, and dwarf plant 9* (Spray et al., 1996; Sakamoto et al., 2004; Cui et al., 2012)*.* Studies have also revealed that jasmonic acid (JA) can affect plant height *via* complex phytohormone crosstalk with GA and auxin (Ren et al., 2021)^.^ Furthermore, NAC transcription factors were shown to enhance maize stalk lodging resistance by directly regulating genes involved in cell wall biosynthesis (Ren et al., 2020). However, the molecular mechanisms regulating maize stalk lodging are still not yet clear.

Recent advancements in next-generation sequencing technologies such as genomics, transcriptomics, proteomics, and metabolomics have allowed fully quantitative gene expression analyses of gene models to be performed. Especially, the transcriptome analysis using RNA-Seq has been a powerful high-throughput tool for the detection of dynamic gene expression profiles and has been widely used to reveal plant–environment interactions (McGettigan, 2012). Compared to traditional sequencing methods, RNA-seq technologies offer several key advantages, such as low cost, high throughput, and high sensitivity. Furthermore, RNA-Seq provides us convenience in detecting transcriptomic changes under certain environments and enables us to identify gene expression profiles in a specific manner (Ren et al., 2021). Meanwhile, metabolomic profiling is being used to detect the differential abundance of metabolites at a global level and also provides useful contributions to molecular genetics studies (Zhao et al., 2019). At present, an integrated analysis combining RNA-Seq and metabolomics can be effective in revealing the molecular mechanisms of maize stem lodging resistance.

In the current study, to better understand stalk lodging resistance of maize, the third stem internode of each of the three diverse maize hybrids (highly resistant JNK738, mildly resistant JNK728, and lowly resistant XY335) was used to perform RNA-Seq transcriptome and metabolome analyses at the tasseling (10 days to silking, DTS) stage. Additionally, for functional analysis, the identified differentially expressed genes (DEGs) and differentially changed metabolites (DCMs) were examined by gene ontology (GO) and metabolic pathway enrichment analyses, respectively. Furthermore, some physiological indices were evaluated to complement RNA-seq and metabolomics approaches in elucidating plants’ stalk lodging tolerance in maize. Our findings broaden our understanding of the molecular mechanisms underlying maize stem lodging resistance, which could potentially open new avenues for breeding maize cultivars with improved lodging resistance.

## Materials and methods

### Plant materials and experimental design

The maize hybrid cultivars diverse in stalk lodging resistance (highly resistant JNK738, mildly resistant JNK728, and lowly resistant XY335) were used in this study. The seeds of these cultivars were provided by the College of Agriculture and Forestry, Hebei North University (Zhangjiakou, China). The seeds were sown in Shalingzi Experimental Station, Zhangjiakou, China (115°05′E, 40°6′N), in 2019. The experiment was set up in a randomized complete block design, with each hybrid cultivar replicated three times. The planting density was 60,606 plants ha^−1^, with 0.55 m * 0.30 m plant spacings. Samples for transcriptomic analysis were collected from the third stem internode above the ground (Xue et al., 2016) of each of the three hybrid cultivars at the 10 DTS stage (described as the stage during tasseling but pre-silk emergence, that is, 10 DTS) (Liu et al., 2020). Five plants were pooled together for a replicate, and each sample had three technical replicates. At every sample collection, we ensured that the samples were immediately liquid nitrogen frozen and stored at extremely low temperatures (-80°C) for further analysis.

### Phenotypic and physiological parameters measurement

The physiological characteristics of the stems of the three hybrid cultivars at the tasseling (10 DTS) stage were determined. The third stem internodes were randomly selected as the material to make about 20 µm thick slices. Each measurement had three technical replications. Then, the stem microstructure was observed and photographed using an Olympus BX51 microscope (Olympus China Co., Ltd., Beijing, China). The RPR and SCS of the third stem internode from each hybrid were tested by stem strength tester YYD-1 (Zhejiang TOP Instrument Co., Ltd., Hangzhou, China). The lignin content of stems was estimated by colorimetry. The phenylalanine ammonia lyase (PAL) activity in the samples was determined using the method developed by Zhang et al. (Okuno et al., 2014). For PAL activity, each sample had three biological replicates, and each measurement had three technical replicates.

### Total RNA extraction, cDNA library construction, and transcriptome analysis

The stalk samples were sent to Wuhan Metware Biotechnology Co., Ltd. (Wuhan, China) for RNA isolation, cDNA library construction, and RNA-Seq. In brief, total RNA was isolated using Trizol reagent (Invitrogen, Carlsbad, CA, United States). Then, the RNA quantity and quality were checked using NanoDrop 1000 (NanoDrop Technologies Inc, Wilmington, DE, United States) and on 1% agarose gels, respectively. The cDNA libraries were generated using NEBNext® Ultra™ RNA Library Prep Kit for Illumina® (NEB, United States) following the manufacturer’s recommendations and then sequenced on the Illumina HiSeq 6000 using the paired-end technology.

### Sequencing reads processing, genome mapping, and gene expression quantification

Raw data (raw reads) of FASTQ format generated by the Illumina HiSeq 6000 system were initially processed through in-house Perl scripts. After removing the low-quality reads, clean reads were mapped onto the maize reference genome (B73 RefGen_v4), allowing large gaps of up to 50 kb to span introns using spliced aligner Tophat 2.0.12 software (Dhindsa et al., 1981). Only reads with a perfect match or one mismatch were further analyzed and annotated based on the reference genome. Additionally, the read numbers mapped to each gene were counted using HTSeq v 0.6.1 software. Quantification of gene expression levels was estimated by fragments per kilobase of transcript per million fragments mapped (FPKM). Gene functions were annotated against the following public databases: non-redundant protein sequence database (Nr), Cluster of Orthologous Groups (COG), Swiss-Port, Kyoto Encyclopedia of Genes and Genomes (KEGG), and Gene Ontology (GO) using BLAST (basic local alignment search) search program.

### Differentially expressed genes detection and functional enrichment analysis

Differential expression analysis was performed using the DESeq R package (1.10.1) (Wang et al., 2019). A differential expression analysis between treatments was performed using the ratio of FPKM values, and the resulting *p-values* were corrected for multiplicity using the Benjamini and Hochberg method (Trapnell et al., 2010). In this study, genes with fold change (FC) ≥ 2 and *Q-value* < 0.05 were assigned as differentially expressed.

For the functional annotation of identified differentially expressed genes (DEGs), GO enrichment analysis was conducted using the Blast2GO tool (http://www.blast2go.com). DEGs were assigned to various metabolic pathways using KOBAS (http://kobas.cbi.pku.edu.cn). The significant KEGG pathway enrichment analysis was conducted by the hypergeometric test (*Q-value* < 0.05). The DEGs with similar expression patterns were identified by K-means clustering using the R package.

### Expression validation of differentially expressed genes with quantitative real-time PCR

To generate a cDNA template, 1 µg of total RNA which had been sent back from Shanghai Majorbio Bio-pharm Technology Co. Ltd. was reverse-transcribed in a total volume of 20 μl, using HiFiscript cDNA Synthesis Kit (CWBIO, Beijing, China) according to the manufacturer’s instructions. To confirm the sequencing results, a total of 20 genes were chosen according to their functions, and specific primers for qRT-PCR analysis were designed using Premier 5 Designer software. The qRT-PCR was conducted using 2 × Fast Super EvaGreen ® qPCR Mastermix (US Everbright Inc, Suzhou, China) on a C1000 (CFX96 Real-Time System) Thermal Cycler (Bio-Rad). Each total 20 µl qRT-PCR reaction mixture was formulated to include 1 µl of templated cDNA, 1 µl of reverse and forward primer (50 pmol), 10 µl qPCR master mix, and 7 µl ddH_2_O. A stable and constitutively expressed maize gene GAPDH (Accession No. X07156) was used for housekeeping. Three biological replicates were used for each sample, and the experimental data were computed using the 2^−ΔΔCT^ method (Anders et al., 2015).

### Metabolites sample preparation and extraction for GC/MS

The freeze–dried stalks were crushed using a mixer mill (MM 400, Retsch) with a zirconia bead for 1.5 min at 30 Hz. 100mg powder was weighted and extracted overnight at 4°C with 0.6 ml 70% aqueous methanol. Following centrifugation at 10, 000 g for 10 min, the extracts were absorbed (CNWBOND Carbon-GCB SPE Cartridge, 250 mg, 3 ml; ANPEL, Shanghai, China, www.anpel.com.cn/cnw) and filtrated (SCAA-104, 0.22 μm pore size; ANPEL, Shanghai, China, http://www.anpel.com.cn/) before UPLC-MS/MS analysis.

### UPLC conditions

The sample extracts were analyzed using a UPLC-ESI-MS/MS system (UPLC, Shim-pack UFLC SHIMADZU CBM30A system, www.shimadzu.com.cn/; MS, Applied Biosystems 4500 Q TRAP, www.appliedbiosystems.com.cn/). The analytical conditions were as follows. UPLC: column, Agilent SB-C18 (1.8 µm, 2.1 mm*100 mm) and the mobile phase consisting of solvent A (pure water with 0.1% formic acid) and solvent B (acetonitrile). Sample measurements were performed using a gradient program that employed the starting conditions of 95% A and 5% B. Within 9 min, a linear gradient to 5% A, 95% B was programmed, and a composition of 5% A, 95% B was kept for 1 min. Subsequently, a composition of 95% A and 5.0% B was adjusted within 1.10 min and kept for 2.9 min. The column oven was set to 40 °C. The injection volume was 4 μl. The effluent was alternatively connected to an ESI-triple quadrupole-linear ion trap (QTRAP)-M.

### Data analysis and validation for metabolites

LIT and triple quadrupole (QQQ) scans were acquired on a triple quadrupole-linear ion trap mass spectrometer (Q TRAP), API 4500 Q TRAP UPLC/MS/MS system, equipped with an ESI turbo ion spray interface, operating in positive and negative ion mode and controlled by Analyst 1.6.3 software (AB Sciex). The ESI source operation parameters were as follows: ion source, turbo spray; source temperature 550 °C; ion spray voltage (IS) 5500 V (positive ion mode)/-4500 V (negative ion mode); ion source gas I (GSI), gas II(GSII), and curtain gas (CUR) set at 50, 60, and 30.0 psi, respectively; and the collision gas (CAD) was high. Instrument tuning and mass calibration were performed with 10 and 100 μmol/L polypropylene glycol solutions in QQQ and LIT modes, respectively. QQQ scans were acquired as MRM experiments with collision gas (nitrogen) set to 5 psi. DP and CE for individual MRM transitions were done with further DP and CE optimization. A specific set of MRM transitions were monitored for each period according to the metabolites eluted within this period. Metabolites with fold change ≥2 or fold change ≤0.05 were assigned as DCMs.

### Statistical analysis of physiological data

In this study, all statistical analyses were conducted using SPSS v.22.0 statistical software (SPSS Institute Ltd, Armonk, NY, United States), and a general mean ± standard error across repeated measurements was calculated and presented. A two-way analysis of variance (ANOVA) and LSD (least significant difference) tests were used to compare the significance of the differences between the control and treatments. Meanwhile, one-way ANOVA and Duncan’s multiple range comparison tests were used to analyze qRT-PCR data.

## Results

### Mechanical and physiological indices of the three maize hybrids

**FIGURE 1:**
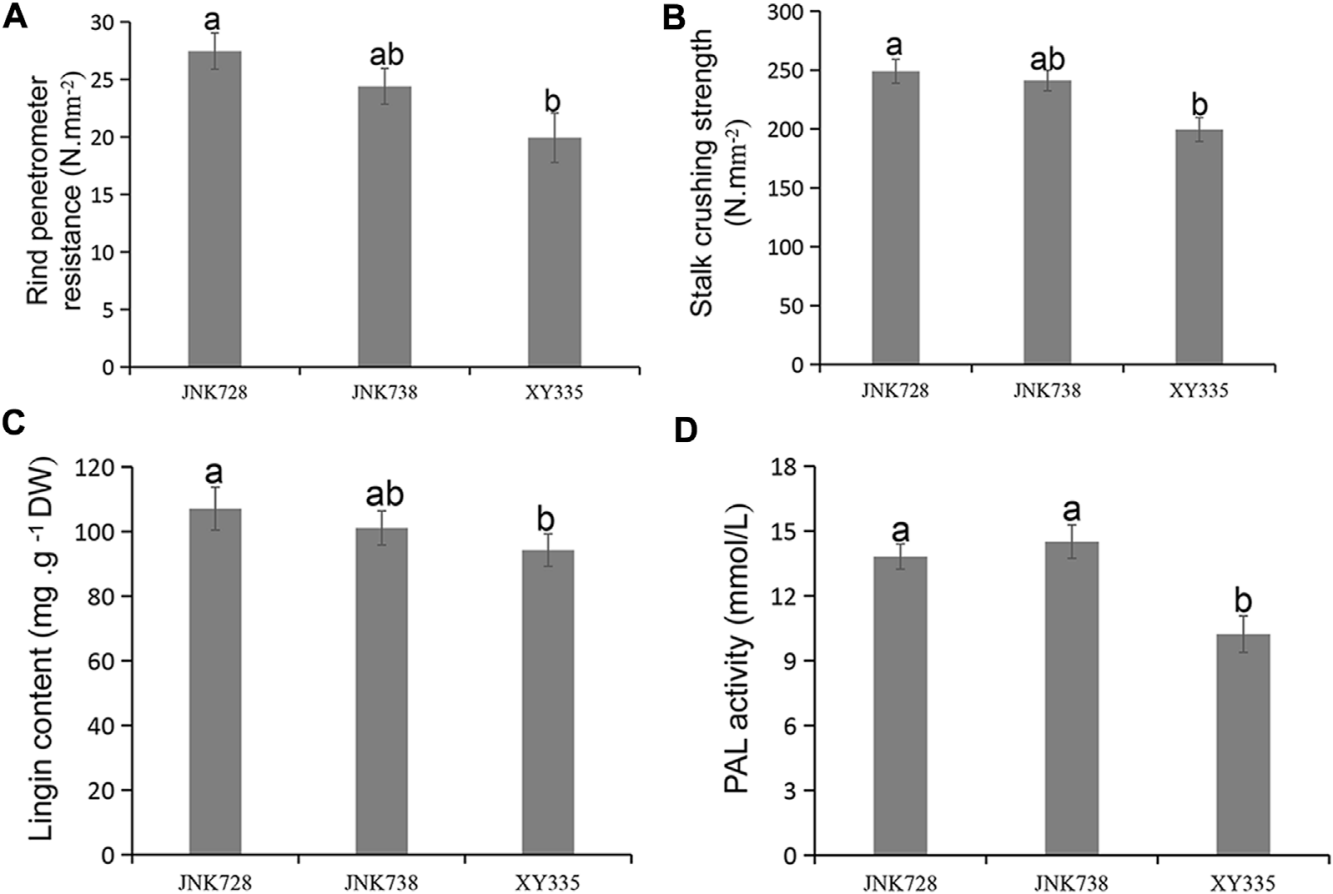
Mechanical index and physiological parameters of the three hybrid cultivars. **(A)** Rind penetrometer resistance, **(B)** stalk crushing strength, **(C)** lignin content, and **(D)** PAL activity. Data are presented as means ± SE (*n* = 3). Different letters above line graphs show a significant difference (*p* ≤ 0.05) among the three hybrids.

At the maize 10 DTS stage, the RPR and SCS of the third stem internode were tested by stem strength tester YYD-1. The average RPR strengths were 19.93 N mm^−2^, 24.40 N mm^−2^, and 27.47 N mm^−2^ for XY335, JN738, and JN728, respectively (Figure 1A). Additionally, the average SCS were 199.60 N mm^−2^, 241.23 N mm^−2^, and 249.07 N mm^−2^ for XY335, JNK738, and JNK728, respectively (Figure 1B). The RPR and SCS showed significant differences between lowly resistant XY335 and highly resistant JNK728 (*p* < 0.05).

Lignin is a phenylpropanoid-derived polymer that is specifically deposited in secondary cell walls, where it enhances stem strength (Vanholme et al., 2010). The total lignin content of the three hybrids of the stem was determined by acetyl bromide analysis, and we found that total lignin content significantly differed between highly resistant JNK728 and lowly resistant XY335 cultivars (Figure 1C). However, the mildly resistant cultivar JNK738 did not significantly differ from JNK728 and XY335. We also measured the PAL activity of the third stem internode of each of the three hybrids. The PAL activity did not show a significant difference between highly resistant JNK728 and mildly resistant JNK738; however, the PAL activity of both JKN728 and JNK738 was significantly different from that of lowly resistant XY335 (Figure 1D).

### Vascular bundle characteristics of the three maize hybrids

**FIGURE 2:**
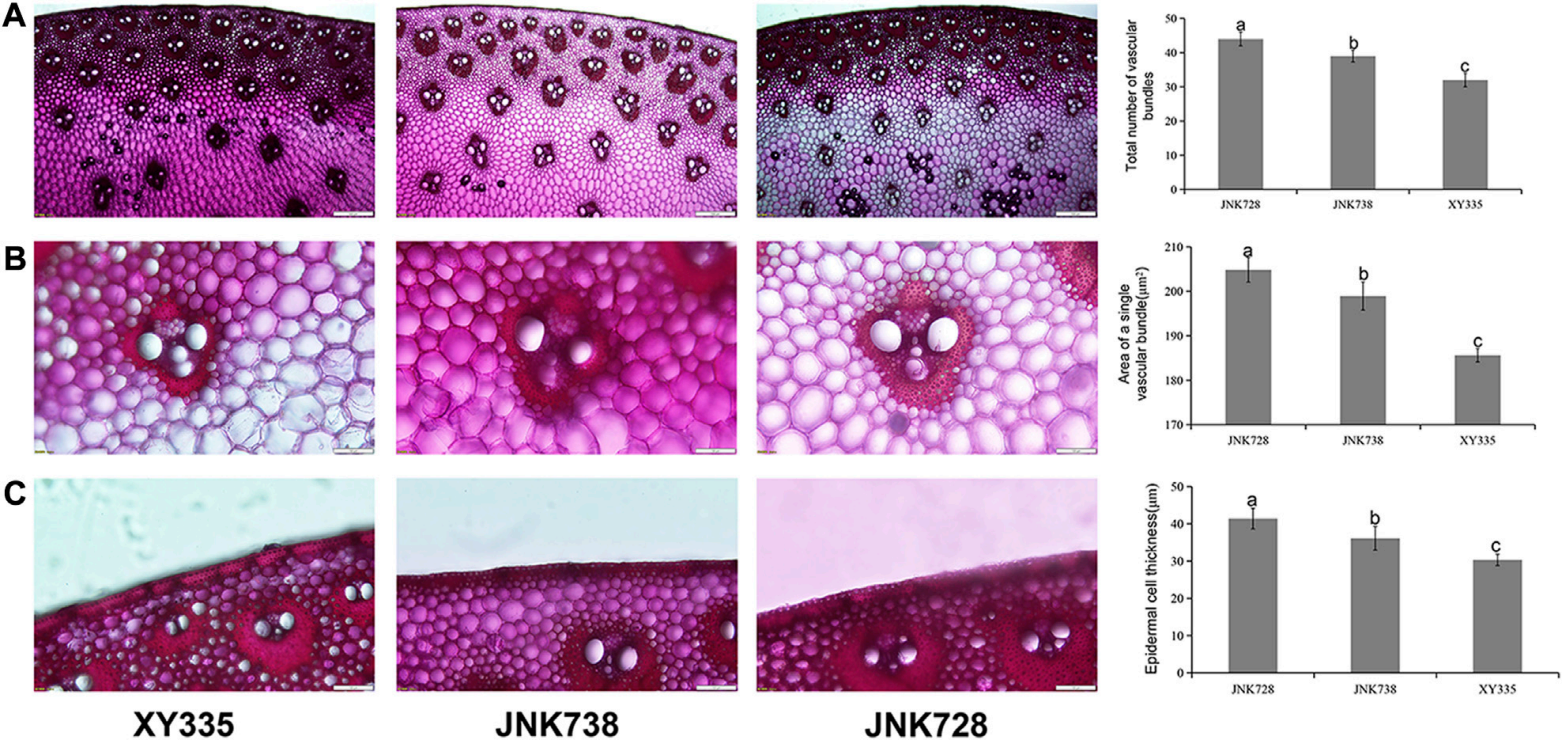
X-ray microcomputed tomography images and vascular bundle traits of the three hybrid cultivars. **(A)** The total number of vascular bundles of XY335, JNK738, and JNK728, respectively. **(B)** Individual vascular bundle area of XY335, JNK738, and JNK728, respectively. **(C)** Epidermal cell thickness of XY335, JNK738, and JNK728, respectively.

Five vascular bundle characteristics of the three maize hybrids were also measured using an Olympus BX51 microscope scan and feature extraction process. We observed that the total number of vascular bundles on the stalk cross-section of XY335, JNK728, and JNK738 was 32, 39, and 44, respectively (Figure 2). Individual vascular bundle area was least in XY335 cultivar and largest in JNK728 cultivar (Figure 2). The order of the thickness of the sclerenchumatous hypodermis layer was JNK728 > JNK738 > XY335 (Figure 2). Meanwhile, the abovementioned traits showed significant differences (*p* < 0.05). Taken together, the mechanical index and vascular bundle characteristics confirmed the varying stalk lodging resistances of the three maize hybrids.

### Analysis of RNA-Sequencing and transcriptomes results

RNA isolated from maize hybrid cultivars diverse in stalk lodging resistance was surveyed using next-generation sequencing. In total, nine samples were obtained and categorized into three groups. Then, cDNA libraries were prepared from these three hybrid cultivars and subjected to RNA-Seq profiling on the Illumina HiSeq 6000 platform. The raw sequencing data were deposited into NCBI Sequence Read Archive (SRA, Accession PRJNA858633). After filtering, a total of 59.26 GB clean reads of 150 bp length were obtained. Of these reads, 84.34%–87.28% could be mapped onto unique positions on the maize reference genome (ZmB73_Ref-Gen_V4). The Q30 base percentage and GC percentages exceeded 93.90% and 53.16%, respectively, which met the requirements for further analysis (Supplementary Table S1). Meanwhile, the principal component analysis (PCA) results showed a high correlation between samples and clear separation of each material (Supplementary Figure S1). Furthermore, cluster analysis for nine samples was conducted using the FPKM method. Cluster analysis results presented the same tendency as the PCA results (Supplementary Figure S1). The abovementioned results indicated that our experiment was reproducible and reliable, which could be used for further analysis.

### Gene differential expression analysis

**FIGURE 3:**
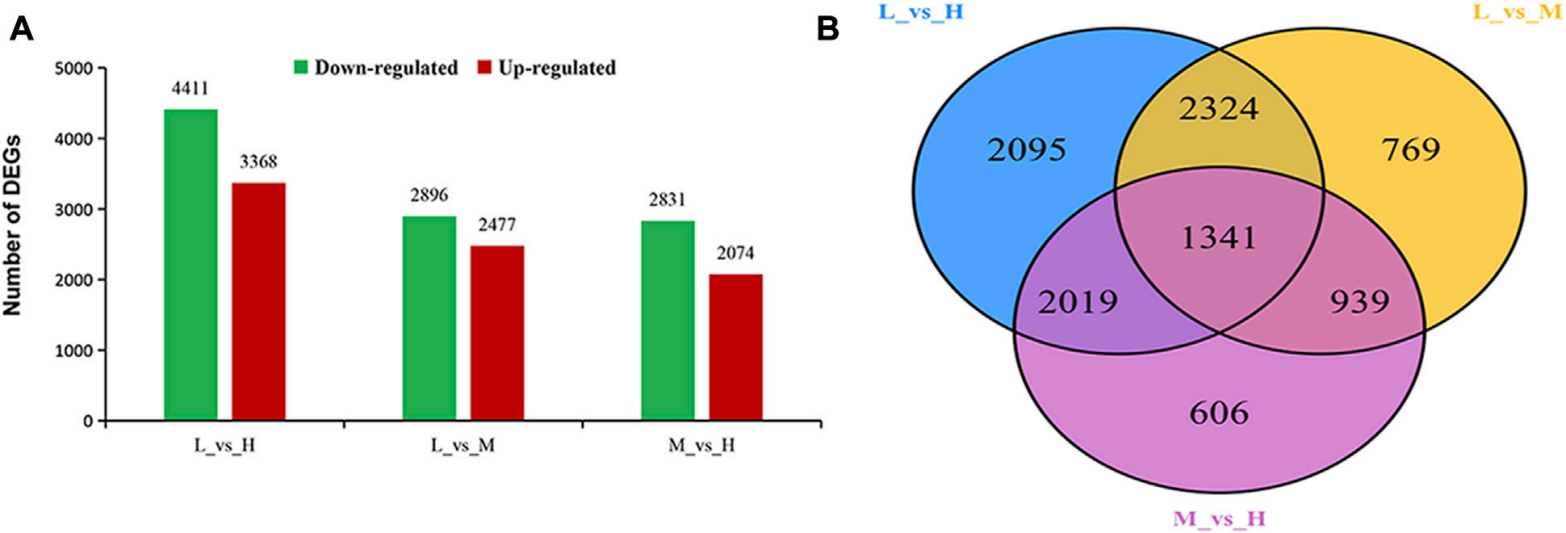
Analysis of differentially expressed genes (DEGs) identified in three experimental comparison groups. **(A)** Total number of DEGs identified in each experimental group. Upregulated means DEGs with increased differential expression. Downregulated means DEGs with decreased differential expression. **(B)** Venn diagram analysis of DEGs observed in the three experimental comparison groups. KEGG Pathways Enrichment Analysis of DEGs.

In order to reveal the gene responses of maize to stalk lodging, we performed a comparative transcriptome analysis to investigate the changes in gene profiles in three maize hybrid cultivars diverse in stalk lodging resistance. We comparatively analyzed the cultivars, and a total of three comparative groups were generated. Based on this analysis, a total of 10,093 DEGs were identified to be differentially expressed among the three comparison groups. We obtained the highest number of DEGs 7779) between XY335 and JNK738 (L_vs._H comparison). Among these DEGs, 4411 were upregulated, and 3368 were downregulated. Meanwhile, 5373 DEGs (2896 upregulated and 2477 downregulated) were identified between XY335 and JNK728 (L_vs._M). Moreover, 4905 DEGs (2831 upregulated and 2074 downregulated) were identified between the JNK728 and JNK738 (M_vs._H) (Figure 3A).

The numbers of DEGs showing overlaps or specific responses in different comparison groups are visualized in the Venn diagram. A great number of DEGs were specifically expressed in diverse groups, including 2095, 769, and 606 DEGs that were specifically expressed in L_vs._H, L_vs._M, and M_vs._H comparison groups, respectively. Meanwhile, we detected 1314 DEGs to be co-expressed in the three comparison groups (Figure 3B).

**FIGURE 4:**
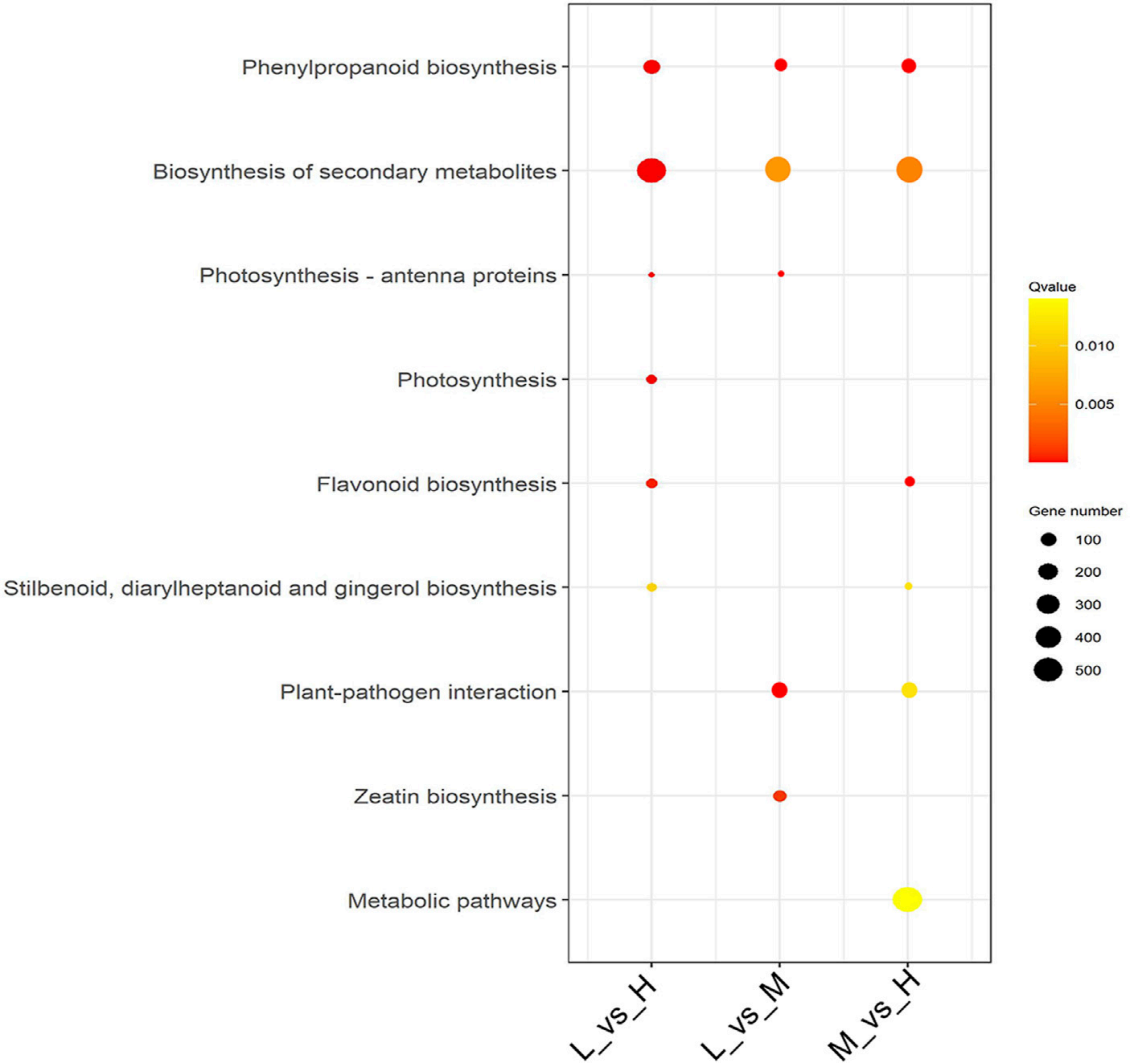
Metabolic pathway enrichment analysis of DEGs. The significance of the enrichment of the KEGG pathway is based on the student’s t-test, *Q-value* < 0.05. Analysis of the dynamic DEGs expression patterns of the three maize hybrid cultivars.

We further analyzed the functional involvement of the DEGs in different pathways by mapping them to the KEGG database. Moreover, we conducted a significant pathway enrichment analysis of those DEGs by hypergeometric test, with pathways of *q*-value < 0.05 considered to be significantly related to maize stalk lodging resistance. The results showed that six metabolism pathways were significantly enriched in both L_vs._H and M_vs._H comparisons, and five metabolism pathways were enriched in L_vs._M comparison (Figure 4). Among them, phenylpropanoid biosynthesis (ko00940) and biosynthesis of secondary metabolites (ko01110) pathways were highly enriched in all the comparisons. Meanwhile, flavonoid biosynthesis (ko00941), stilbenoid, diarylheptanoid, and gingerol biosynthesis (ko00945) pathways were highly enriched in L_vs._H and M_vs._H comparisons; whilst photosynthesis-antenna proteins (ko00196) pathway was dominant in L_vs._H and L_vs._M comparisons.

**FIGURE 5:**
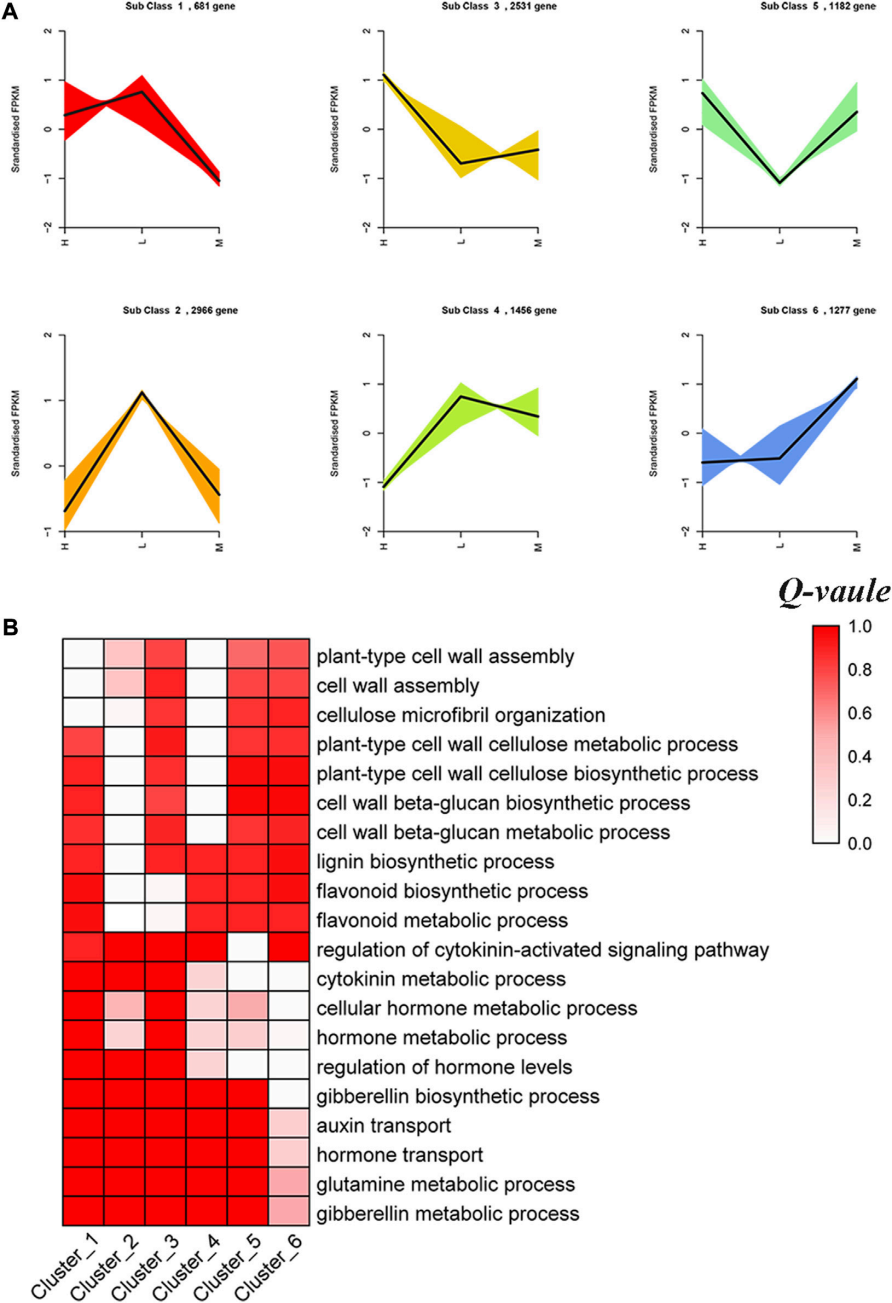
Cluster analysis of stem lodging resistance–related DEGs. **(A)** Six clusters show the analysis results of the gene expression profiles with the K-means algorithm. **(B)** GO enrichment analysis of the DEGs in each cluster. *Q-value* means enrichment factor, where 0.0 is no enrichment and 1.0 is highly enriched. Validation of DEGs by quantitative real-time PCR (qRT-PCR).

To investigate the expression patterns of DEGs identified in the three hybrid cultivars, K-means clustering analysis, which enabled us to observe gene clusters with distinct expression profiles, was performed. Resultantly, a total of 10,093 DEGs were divided into six clusters comprising 682–2966 same expression trend genes (Supplementary Table S2). Cluster 1, cluster 2, and cluster 4 included 681, 2966, and 1456 DEGs, respectively, which all showed high expression in lowly resistant maize hybrid XY335 (Figure 5). GO enrichment analysis of each cluster highlighted vital biological processes enriched by the DEGs with the same expression trend. We observed that cluster 1 was highly enriched with plant-type cell wall assembly (GO:0071668), cellulose microfibril organization (GO:0010215), and cell wall assembly (GO:0070726). Meanwhile, GO terms plant-type cell wall cellulose metabolic process (GO:0052541), cell wall beta-glucan biosynthetic process (GO:0009809), and lignin biosynthetic process (GO:0009809) were prominent in clusters 2 and 4. Additionally, we observed that 2531 DEGs of cluster 3 related to flavonoid biosynthetic process (GO:0009813) and flavonoid metabolic process (GO:0009812) exhibited high expression in highly resistant cultivar JNK728. Moreover, cluster 5 included 1182 DEGs mainly involved in the hormone metabolic process (GO:0042445), regulation of hormone levels (GO:0010817), and cytokinin metabolic process (GO:0009690), and they exhibited high expression in JNK728 and JNK738 but low expression in XY335. We also observed that cluster 6 comprised 1227 DEGs associated with hormone regulation and showed high expression in mildly resistant cultivar JNK738 (Figure 5, Supplementary Table S3).

In order to confirm the accuracy of the RNA-Seq results, we performed a validation experiment by qRT-PCR analysis for three biological replicates. We randomly chose a sample of 20 DEGs, and the gene-specific primers (Supplementary Table S4) were designed using Primer Premier 5.0 software (Premier Biosoft International, Palo Alto, CA, United States). Precisely, the patterns of RNA seq expression of all the DEGs were consistent with the qRT-PCR approach (Supplementary Figure S2A; Supplementary Table S5), with a correlation coefficient (*R* ^2^) of 91.17% (Supplementary Figure S2B). This endorsed our RNA sequencing data as reliable.

### Regulation of lignin-related metabolites of the three maize hybrid cultivars

**FIGURE 6:**
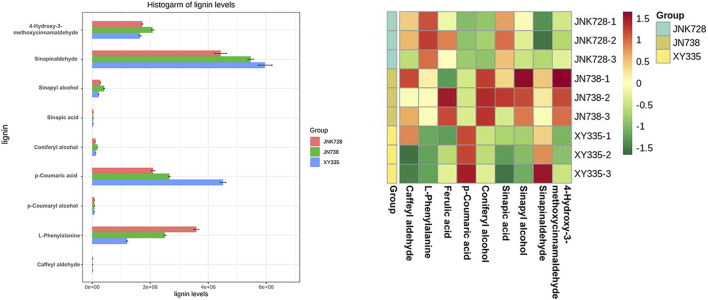
Lignin content quantification in the samples of the three maize hybrid cultivars. The bar graphs on the left show the lignin content of each cultivar, and the plot on the right is the cluster heat map of the lignin content.

Lignin is a secondary metabolite in nature whose content is inferior to cellulose. Lignin is filled in the cellulose framework to enhance the mechanical strength of plants, facilitating the water transport of tissues and resisting the invasion of the adverse external environment (Vanholme et al., 2010). In this research, LC-MS/MS was used to detect 14 intermediates of the lignin pathway and nine metabolites that were detectable in all three maize hybrid cultivars. These metabolites represented differential expression patterns among three maize hybrid cultivars (Figure 6). For example, five metabolites (caffeyl aldehyde, L-phenylalanine, ferulic acid, p-coumaric acid, and sinapic acid) exhibited a similar regulation pattern between JNK728 and JNK738, and three metabolites (coniferyl alcohol, sinapyl alcohol, and 4-hydroxy-3-methoxycinnamaldehyde) showed the same regulation pattern between XY335 and JNK738. In particular, L-phenylalanine was determined as a DAM in both L_vs._H and L_vs._M comparisons, and it exhibited apparent upregulation in these two comparisons. Additionally, p-coumaric acid showed downregulation, which was determined as DCMs in L_vs._H comparison. These two DCMs belonged to phenylalanine metabolism and biosynthesis pathways, as was also supported by the RNA-Seq data. A series of DEGs correlated with both DCMs were related to these two pathways, indicating that these DEGs were valuable potential targets for improving maize stalk lodging resistance since they likely regulate the metabolism of L-phenylalanine and p-coumaric acid.

### Plant hormone–related metabolites regulating maize stalk lodging resistance

Plant hormones refer to the trace amount of organic substances synthesized in plants, usually transported from the synthesis site to the site of action, and produce significant effects on the growth and development of plants (Veldstra, 2011). Based on LC-MS/MS method, eight major classes of phytohormones were quantified; the list of phytohormones, correlated genes, and metabolic pathways is listed in Supplementary Table S6. We found that these pathways were significantly related to maize stem lodging resistance. Key among the observed pathways were those involved in phenylalanine metabolism, tryptophan metabolism, alpha-Linolenic acid metabolism, and plant hormone signal transduction.

As shown in Table 1, genes encoding phenylalanine ammonia lyase (PAL) were differentially expressed in the three comparisons. PAL catalyzes the first step in phenylpropanoid, and gene *LOC103653804* showed decreased expression in all three comparisons. *LOC109946043* was specifically expressed in the comparison of lowly resistant and highly resistant hybrids. Meanwhile, two genes (*LOC100381820* and *LOC100381820*) were specifically differentially expressed in the comparison of mildly resistant and highly resistant hybrids.

**TABLE 1:** Annotation of DEGs associated with plant hormone–related metabolites regulating stalk lodging resistance in maize.

Pathways:Phenylalanine metabolism				
Metabolites:Salicylic acid				
Gene ID	Gene annotation	L_vs._H	L_vs._M	M_vs._H
LOC100273579	Uncharacterized protein	−2.96		−1.99
LOC100279491	Primary amine oxidase precursor	−1.80	−1.69	
LOC100280893	Chemocyanin precursor	−3.01	−1.91	−1.09
LOC100281532	Phenylalanine ammonia-lyase	−5.64		−4.28
LOC100284336	Chemocyanin	−2.71		−2.08
LOC100285115	Uncharacterized protein	−2.21	−1.45	
LOC100384215	Uncharacterized protein LOC100384215 isoform X1	−2.79	−1.06	−1.74
LOC103636788	Nascent polypeptide-associated complex subunit alpha, muscle-specific form	−3.65		−4.52
LOC103641885	Uncharacterized protein	−4.91	−1.06	−3.86
LOC103653804	Phenylalanine ammonia lyase3	−3.94	−1.69	−2.26
LOC109946043	Phenylalanine ammonia-lyase	−3.43		
pal3	Phenylalanine/tyrosine ammonia-lyase	−3.13		−2.62
pco075539	Uncharacterized protein	−1.14		
pco118120	Uncharacterized protein LOC100383438 isoform X1	1.28	1.13	
LOC100381820	Phenylalanine ammonia-lyase		2.91	
LOC100274380	Uncharacterized protein			−3.74
LOC100281042	Phenylalanine ammonia-lyase			1.54
LOC100283222	Chemocyanin precursor			−3.34
LOC100381820	Phenylalanine ammonia-lyase			−3.36
LOC103629504	Uncharacterized protein LOC103629504			−5.28
LOC109943165	Fatty acid amide hydrolase			1.05

The plant hormone signal transduction pathway–related genes encoding auxin, abscisic acid, jasmonates, and salicylic acid were differentially expressed in the three comparisons of stalk lodging resistance. A total of 132, 70, and 86 DEGs were observed in L_vs._H, L_vs._M, and M_vs._H comparison groups, respectively (Supplementary Table S7). Most genes were downregulated in comparison to lowly resistant and highly resistant hybrids, including those encoding AUX/IAA family. Moreover, a series of TFs were identified in the three comparisons, including MYB, bZIP, and WRYK. Taken collectively, these changes in key genes could affect maize stalk lodging resistance.

## Discussion

Maize plays an important role in China’s food security strategy; however, with the increase in maize planting density, the problem of stalk lodging is increasingly becoming serious and one of the factors limiting yield and quality. Therefore, developing maize cultivars with high stalk mechanical strength and strong lodging resistance is a priority for maize breeders. In this study, to better understand maize stem lodging resistance, third stem internodes of three diverse hybrid cultivars were used to perform RNA-Seq and metabolomic analyses at the 10 DTS stage. In addition to the stalk mechanical index, some physiological indices and vascular bundle characteristics were evaluated to complement RNA-seq and metabolic approaches in elucidating maize stem lodging resistance. Our findings provide further understanding of the biological networks and molecular mechanisms of stalk lodging resistance and potentially open up new avenues for stalk lodging resistance breeding in maize.

### Hybrid cultivars showed differences in the mechanical index and physiological and vascular bundle characteristics

Our experimental findings on mechanical index and physiological and vascular bundle characteristics showed that the three hybrids performed differently in terms of stalk lodging resistance. The RPR and SCS analysis results showed significant differences between lowly resistant XY335 and highly resistant JNK728. However, no significant difference was observed between highly resistant JNK728 and mildly resistant JNK738 and between mildly resistant JNK738 and lowly resistant XY335 (Figures 1A and B). Rind strength is a vital predictive phenotype of stalk lodging resistance due to its relationship with stem lodging and grain yield (Feng et al., 2010; Liu et al., 2020). RPR, as an important measurement index, can efficiently and accurately evaluate stalk strength to enhance the lodging resistance of breeding lines (Jampatong et al., 2000). RPR has been reported to be significantly and positively related to stem lodging resistance in previous studies (Sibale et al., 1992; Zhao et al., 2013).

The content of lignin is associated with stem rigidity because lignin is only deposited in the secondary cell wall, which influences the structural integrity of the cell wall and stiffness of the stem (Kumar et al., 2020). Our investigation of the lignin content of the three hybrid cultivars revealed that highly resistant JNK728 always accumulated greater amounts of lignin than the other two cultivars in response to stalk lodging (Figure 1C). In a study on wheat, the stalk lignin content was significantly related to stalk strength and lodging resistance (Peng et al., 2014). PAL catalyzes the first step in a series of enzymatic reactions generating lignin monolignols from phenylalanine (Vanholme et al., 2008). Our results showed that PAL activity did not show a significant difference between highly resistant JNK728 and mildly resistant JNK738, but PAL activity of both JNK728 and JNK738 was significantly different to that of lowly resistant cultivar XY335 (Figure 1D).

Additionally, by scanning stem cross-sections using simple and microcomputed tomography techniques, we observed that the density of the vascular bundles and the individual vascular bundle area in the highly resistant JNK728 were greater as compared to JNK738 and XY335 (Figure 2). The number of vascular bundles and thickness of the sclerenchumatous hypodermis layer determine the compressive strength of the maize stalk (Trapnell et al., 2010), and the current study results suggested that the more the number of vascular bundles and the thicker the epidermal cells, the greater the stalk lodging resistance. Taken together, our results showed that the three maize hybrid cultivars varied considerably in their phenotypic responses to stalk lodging, with JNK728 being comparably more lodging resistant than JNK738 and XY335, probably due to its higher mechanical strength and stem strength and greater cell wall intensity.

### Lignin biosynthesis plays important roles in maize stalk lodging resistance

The maize stems’ transcriptomes revealed changes in major metabolic pathways (Figure 4). We observed that phenylpropanoid biosynthesis and biosynthesis of secondary metabolites pathways were highly enriched in L_vs._H, L_vs._M, and M_vs._H comparisons. In addition, integrated metabolome and transcriptome analyses revealed that the nine metabolites associated with lignin were differentially changed in the three hybrid cultivars, and the phenylalanine metabolism pathway was observed to be significantly related to maize stem lodging resistance. The phenylpropanoid metabolism pathway serves to offer these secondary metabolites, which then helps to improve stalk lodging resistance. The phenylpropanoid pathway is the main pathway for lignin synthesis. It is well known that lignin content is involved in stem rigidity, and it has been confirmed that it is higher in strong stems than in weaker ones (Ma, 2009). In our study, the genes related to lignin biosynthesis were observed, including *PAL*, *4-coumarate-CoA ligase* (*4CL*) and *CAD*. PAL catalyzes the first step in a series of enzymatic reactions generating lignin monolignols from phenylalanine (Vanholme et al., 2008). In wheat, COMT is the key enzyme for lignin biosynthesis and critical for the formation of stalk strength. Previously, PAL, 4CL, CAD, and COMT were reported to play vital roles in lignin biosynthesis (Zheng et al., 2016). The DEGs correlated with both DCMs were related to phenylpropanoid biosynthesis, indicating that these DEGs were valuable potential targets for improving maize stalk lodging resistance since they likely regulate the metabolism of lignin accumulation which is a vital factor affecting stem strength.

### Specific expression patterns of the stalk lodging resistance genes in maize

To better understand the regulation patterns of genes identified in each cultivar, K-means analysis was conducted to cluster those DEGs into clear and distinct expression profiles. We identified that most clusters were associated with stalk lodging resistance (Figure 5). Those clusters offered ample evidence of the involvement of DEGs in molecular modulation of lodging resistance in different cultivars. Particularly, cluster 1, cluster 2, and cluster 4 comprised 681, 2966, and 1456 DEGs, respectively, which enriched to cell wall assembly and lignin biosynthetic process showing high expression in lowly resistant maize hybrid XY335. The cell wall is a strong fibrillar network which offers mechanical support to the entire plant. The fabric and structure of the cell wall play important roles in the stalk strength and stalk lodging resistance of plants (Jian et al., 2006). In this current study, some genes related to the cell wall were identified in those clusters, including expansion protein (EXP) and xyloglucan endotransglucosylase/hydrolase proteins (XETs). EXPs are encoded by a super family of genes, which can promote cell wall loosening and extension. In rice, the EXP genes have been implicated in plant growth promotion (Showalter, 1993). XETs cleave xyloglucan polymers and ligate the newly generated reducing ends to other xyloglucan chains, thereby playing critical roles in cell wall plasticity and cell elongation (Pan et al., 2019). The XET genes are potentially involved in various aspects of plant growth, development and external stimulus where the process of cellular expansion is required.

Meanwhile, we observed that 2531 DEGs of cluster 3 (involved in flavonoid biosynthetic process and flavonoid metabolic process) exhibited high expression in the highly resistant hybrid JNK728. However, genes associated with flavonoid biosynthesis showed downregulation in soybean stem during lodging (Liu et al., 2019). A coordinated reaction of the genes and pathways related to secondary metabolite biosynthesis is, thus, considered important to enhance stalk lodging resistance. Additionally, the DEGs related to the hormone metabolic process (cluster 5) exhibited high expression in JNK728 and JNK738 but low expression in XY335. Furthermore, we also observed that cluster 6 comprised 1227 DEGs associated with plant hormone regulation which showed high expression in the mildly resistant hybrid JNK738. The genes encoding ABA, GA, IAA, and JA, were identified in cluster 5 and cluster 6. Previously, the mutation of *sd1,* encoding a *GA* biosynthetic factor, decreased clum strength by reducing culm diameter and thickness (Vanholme et al., 2010). Taken together, these results showed that stalk lodging resistance genes exhibited different expression patterns among the three maize hybrid cultivars; however, genes related to secondary metabolites biosynthesis – lignin, flavonoids, and cell wall were vital in enhancing stalk lodging resistance.

## Conclusion

In the present study, we have applied an integrated transcriptome, metabolome, and physiological analyses approach to decipher the differential responses of three maize hybrids with diverse stalk lodging resistance at the tasseling stage. By RNA-seq analysis, a total of 10,093 DEGs were identified from the comparison of three varieties in pairs. In L_vs._H and M_vs._H comparisons, the DEGs predominantly associated with phenylpropanoid and biosynthesis of secondary metabolites pathways were significantly enriched. Meanwhile, K-means analysis clustered those DEGs into clear and distinct expression profiles in each hybrid cultivar. DEGs were highly enriched in the cell wall assembly, lignin biosynthetic process, and hormone metabolic process and several functional and regulatory genes were identified in the special clusters related to lodging resistance. Integrating metabolome and transcriptome analyses revealed important lignin-associated DCMs that regulate stalk lodging resistance, including L-phenylalanine. Moreover, several hormone signal transduction pathway–related genes were differentially expressed and were involved in stalk lodging resistance. Furthermore, our physiological analysis of stalks indicated that the high stalk lodging resistance of JNK728 may be due to its higher mechanical strength and greater cell wall intensity. More strikingly, stalk lodging resistance candidate genes were implicated in the cell wall assembly, lignin biosynthetic process, phenylpropanoid biosynthesis, and hormone metabolic processes. Our results provide an elaborate understanding of the molecular networks mediating maize stalk lodging resistance and offer a fundamental basis for further targeted research studies such as cloning and downstream analysis of the identified specific individual genes.

## Data Availability

The datasets presented in this study can be found in online repositories. The names of the repository/repositories and accession number(s) can be found at: https://www.ncbi.nlm.nih.gov/, PRJNA858633.
