# Supplementary material for: Integration of transcriptome and metabolome analyses reveals key lodging-resistance-related genes and metabolic pathways in maize
Source: Front Genet. 2022 Oct 10;13:1001195. doi: 10.3389/fgene.2022.1001195 (PMC9588961; doi:10.3389/fgene.2022.1001195)
Supplement: Supplementary file 1 [file DataSheet1.zip › Supplementary Table 1.doc]

Supplementary Table 1. The summary of RNA-Seq data

| **Sample** | **Raw Reads** | **Clean Reads** | **Q30(%)** | **GC Content (%)** | **Unique mapped** | **Multi mapped** |
| --- | --- | --- | --- | --- | --- | --- |
| JN738-1 | 45628206 | 43858376 | 94.36 | 53.69 | 38079538(86.82%) | 2519952(3.37%) |
| JN738-2 | 49113472 | 46927518 | 94.46 | 53.78 | 40671536(86.67%) | 2782255(3.41%) |
| JN738-3 | 47537856 | 45732776 | 93.90 | 53.72 | 39628836(86.65%) | 2690579(3.43%) |
| JNK728-1 | 46743554 | 44778802 | 94.64 | 53.21 | 38131199(85.15%) | 2833048(3.58%) |
| JNK728-2 | 42213792 | 40278042 | 93.90 | 53.24 | 34173234(84.84%) | 2543399(3.56%) |
| JNK728-3 | 45140086 | 42175780 | 94.60 | 53.16 | 35820137(84.93%) | 2740012(3.64%) |
| XY335-1 | 45920308 | 44134008 | 94.30 | 54.57 | 38518679(87.28%) | 2222552(3.04%) |
| XY335-2 | 44670476 | 42529790 | 94.55 | 54.84 | 37079717(87.19%) | 2186084(3.04%) |
| XY335-3 | 46723572 | 44596496 | 94.02 | 55.08 | 38796470(86.99%) | 2319389(3.06%) |
